# A Machine Learning Based Intrusion Detection System for Mobile Internet of Things

**DOI:** 10.3390/s20020461

**Published:** 2020-01-14

**Authors:** Amar Amouri, Vishwa T. Alaparthy, Salvatore D. Morgera

**Affiliations:** 1 Department of Electrical Engineering, University of South Florida, Tampa, FL 33620, USA; aamouri@mail.usf.edu; 2 Department of Electrical and Computer Engineering, Duke University, Durham, NC 27708, USA; vishwa.alaparthy@duke.edu

**Keywords:** intrusion detection systems, WSN, IoT, random forest, AMoF, linear regression

## Abstract

Intrusion detection systems plays a pivotal role in detecting malicious activities that denigrate the performance of the network. Mobile adhoc networks (MANETs) and wireless sensor networks (WSNs) are a form of wireless network that can transfer data without any need of infrastructure for their operation. A more novel paradigm of networking, namely Internet of Things (IoT) has emerged recently which can be considered as a superset to the afore mentioned paradigms. Their distributed nature and the limited resources available, present a considerable challenge for providing security to these networks. The need for an intrusion detection system (IDS) that can acclimate with such challenges is of extreme significance. Previously, we proposed a cross layer-based IDS with two layers of detection. It uses a heuristic approach which is based on the variability of the correctly classified instances (CCIs), which we refer to as the accumulated measure of fluctuation (AMoF). The current, proposed IDS is composed of two stages; stage one collects data through dedicated sniffers (DSs) and generates the CCI which is sent in a periodic fashion to the super node (SN), and in stage two the SN performs the linear regression process for the collected CCIs from different DSs in order to differentiate the benign from the malicious nodes. In this work, the detection characterization is presented for different extreme scenarios in the network, pertaining to the power level and node velocity for two different mobility models: Random way point (RWP), and Gauss Markov (GM). Malicious activity used in the work are the blackhole and the distributed denial of service (DDoS) attacks. Detection rates are in excess of 98% for high power/node velocity scenarios while they drop to around 90% for low power/node velocity scenarios.

## 1. Introduction

Mobile adhoc networks (MANETs), wireless sensor networks (WSNs), and Internet of Things (IoT) are a class of networks that deploy low resources nodes and the nodes that require rapid deployment. The goal is to develop an intrusion detection system (IDS) capable of dealing with such constraints. These IoT devices not only help in transmitting and receiving data, but also connect various devices to the Internet. These devices can be mobile or stationary depending on the application they are supposed to be used for. MANETS and mobile WSNs are the type of IoT networks, we are attempting to secure in this work. Machine learning and artificial intelligence-based IDSs were studied extensively during the last decade. Various machine algorithms were explored such as: Neural networks [1] and its newer version, deep learning [2], support vector machines (SVM) [3], decision trees [4], k-NN clustering [5], and Naïve Bayes [6]. However, a study presented by [7] shows several advantages for using random forest when it comes to the complexity, accuracy, and memory usage. The rationale for using random forest as a core algorithm in our previous cross layer-based IDS is its suitability for the resource restrictions inherent in the afore mentioned networks [8].

Apart from machine learning there are other techniques which have been employed to build an intrusion detection system. A broader classification of these techniques, segregates IDSs as anomaly-based IDSs, signature based, and specification based IDSs. Markov models and hidden Markov models [9] have been the crux of the many IDSs that have proved efficient. Swarm intelligence [10] has also been used in order to try and decrease the training time of the IDS. A considerable number of hybrid schemes [11] are also employed, which proved more effective than the conventional models. In addition, there has been an alternate field of study [12], which take the human immune system (HIS) as an inspiration and derives an IDS for IoT networks. IDSs engineered [13,14] from HIS are commonly based on three different immune theories namely danger theory, negative selection, and clonal selection.

In this paper, a two-stage cross layer-based IDS is presented. Stage one is composed of five dedicated sniffers (DSs) which collect data from MAC and network layer. It is then fed to a random forest classifier, mounted on each DS, which generates a quantity known as correctly classified instances (CCIs). These CCIs are fed to a super node (SN) which is stage two. It performs a sliding window algorithm on all the CCIs collected from different DSs. This process calculates a parameter which we call the accumulated measure of fluctuation (AMoF). In addition, the SN performs an iterative linear regression process on the AMoF points. A detection threshold is chosen to separate the boundaries between the malicious and normal nodes. A key idea used in the proposed IDS, is that the variability of CCIs in the smaller size population, which represents the number of malicious nodes in the network is smaller than the variance of the larger size population, which represents the number of normal nodes in the network.

In this paper, we expand the previous work [8,15] and test the proposed architecture under a wide range of malicious activities such as blackhole and DDoS (flooding attack) and under other scenarios, such as mobility models. The proposed scheme is tested under two different mobility models; random way point (RWP) and Gauss Markov models (GM). The latter is used to add a more realistic mobility model which consists of a temporal correlation for nodes position based on certain parameters in this model.

This paper is divided into the following sections: Section 2 presents a brief survey of the related work. The system architecture of a multilevel detection approach utilizing random forest and linear regression is described in Section 3, while Section 4 presents a brief introduction about the blackhole attack and flooding attacks adopted in this paper. In Section 5, the experimental setup is explained in detail. Results and discussion are provided in Section 6. Finally, Section 7 concludes this paper.

## 2. Related Work

In this section, a simple survey for major machine learning techniques used in IDS for MANETS, WSN, and IoT is presented. The main material is taken from A. Amouri dissertation [16].

Deng et al. [17] proposed an IDS based on SVM classification algorithm for two types of IDS architecture, distributed and hierarchal. Detection rates well above 90% were achieved by using biasing in the feature selection.

An ensemble-based IDS for MANETs was proposed by Cabrera [18,19], where a three-level hierarchical system for data collection, processing, and transmission was described. The anomaly index at each level is calculated and the final decision is performed at the highest hierarchy. The authors used the receiver operating characteristic (ROC) curve and the corresponding area under curve (AUC) to characterize the performance of their proposed scheme. A C4.5 decision tree in conjunction with the CFA algorithm was used for detection purposes.

A dynamic learning method to detect blackhole attacks on AODV-based MANETs is proposed by Kurosawa et al. [20]. A dynamic training method in which the training data is updated at regular time intervals serves as the main concept for detecting malicious activity in the network. A simple clustering algorithm is used to identify the malicious nodes. Detection rates versus node mobility are used for performance characterization, ranging from 70% to 84% for node mobility between 0 and 20 m/s.

In the proposed scheme by Bose et al. [21], a Bayesian classification algorithm, Markov chain construction algorithm and association rule mining algorithm for anomaly detection in MAC, routing and application layer, respectively for effective intrusion detection has been deployed. Detection rates of 94.33% and 0.8% false positive rate (FPR) were achieved at the global integration module.

An IDS based on neural networks and watermarking techniques was presented by Mitrokotsa and Komninos [22]. Detection rates around 90% with high false alarms (more than 20%) are reported. The detection rates were shown to be higher for longer periods of pause times.

Mitrokotsa et al. [23] analyzed the performance of well-known five supervised classification algorithms (the Naïve Bayes model, the linear model, the Gaussian mixture model, multilayer perceptron, and (SVM) model) used as a detection technique in detection engines for MANETs. Their results showed that the Naïve Bayes classifier has the poorest performance while the best performance is achieved with the multilayer perceptron classifier.

Azmoodeh and Choo [24], used the deep eigenspace learning for malware detection in “Internet Of (Battlefield) Things Devices”. The accuracy, precision, recall, and F-measure are: 99.68%, 98.59%, 98.37%, and 98.48%, respectively.

Doshi et al. [25] tested five machine learning algorithms to distinguish normal IoT packets from DoS attack packets. The algorithms are: (1) K-nearest neighbors “KDTree” algorithm; (2) support vector machine with linear kernel (LSVM); (3) decision tree using Gini impurity scores; (4) random forest using Gini impurity scores; (5) neural network. The random forest showed the best results among the tested classifiers for the precision, recall, F1, and accuracy tests.

Thamilarasu and Chawla [26] proposed a deep learning-based IDS for IoT, the following attacks were investigated: Blackhole attack, opportunistic attack, DDoS attack, Sinkhole attack, Wormhole attack, the TPR are 96.4%, 98%, 98.7%, 99%, 98%, respectively.

## 3. System Architecture

**Figure 1:**
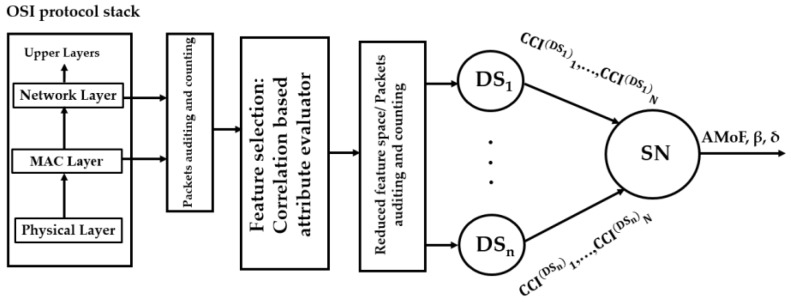
A two stage cross layer IDS.

In this section, the system architecture for the cross-layered IDS is presented. The IDS is composed of two stages of detection as shown in Figure 1. At stage one, the dedicated sniffers (DSs) collect data, which is a packet count from both MAC and network layers as shown in Table 1. These are first-hand features collected through promiscuous mode which reduces the misleading data collected by direct reporting from the nodes themselves [27]. We use five DSs in this paper and they monitor an area of 1000 m^2^.

Every DS generates a CCI per reporting time (*Tr*). There are *N* instances of *Tr* as shown in Figure 1. Once two CCI samples are collected by the SN at stage two from each DS, an iterative process using linear regression which calculates the slope $\left(\beta_{1}\right)$ and the threshold (δ) is performed as shown in Algorithm 1. 

Linear regression explains the dependency between the dependent variable X and independent variable Y as [28],

(1) $$Y_{i}=\beta_{0}+\beta_{1}X_{i}+\epsilon_{i}$$

where $\beta_{0}\;$ and $\beta_{1}\;$ are the model parameters. The errors $\epsilon_{i}$ are assumed to be independent $N\left(0,\sigma^{2}\right)$. The confidence interval for $\beta_{1}$ is given as

(2) $$b_{1}\pm \frac{t\left(n-2,1-\frac{\alpha }{2}\right)s}{{\left\{\sum {\left(x_{i}-\bar{x}\right)}^{2}\right\}}^{1/2}}$$

where $t\left(n-2,1-\frac{\alpha }{2}\right)$ is the $100\left(1-\frac{\alpha }{2}\right)$ percentage point of a t-distribution with (n−2) degrees of freedom and the residual sum of squares $s^{2}$. Equations (1) and (2), are used in Algorithm 1 to calculate the iterative fitted slope and the confidence interval based on the CCIs points collected from different DS regarding any node under test (NUT). A more detailed explanation about how the confidence interval is used in the detection characterization is presented in the Results Section 6.

It is important to mention that the values of the CCIs at the first stage does not yield information regarding the state of the tested nodes whether it is malicious or benign. It is the variability of the CCIs collected at the SN based on the sliding window-based algorithm as shown in Algorithm 1, that make the distinction between the state of two nodes feasible.

**Algorithm** **1** Calculating the AMoF, fitted slope, confidence intervals, and detection threshold1: **Input:**
$CCI^{\left(DS_{m}\right)}{}_{1}$,………,$CCI^{\left(DS_{m}\right)}{}_{N}$, ∀ m ∈n2: **Output:** AMoF, fitted slope (β), detection threshold (δ)3: **At the super node**4: ∀ node∈NUT where the number of elements in **NUT** = *l*5: Receive $CCI^{\left(S_{n}\right)}{}_{1}$,………, $CCI^{\left(S_{n}\right)}{}_{N}$ S.T $CCI^{\left(NUT\right)}is\;N\times n$6: Initialize $Temp^{\left(S_{j}\right)}$, Norm_$Temp^{\left(S_{j}\right)}{}_{i}$, $AMoF^{\left(S_{j}\right)}{}_{i}$7: **For**
*i* = 1 to N
**do**8: **For**
*j* = 1 to *n*
**do**9: $TEMP_{i}{}^{\left(S_{j}\right)}$←$\left|CCI_{i+1}{}^{\left(S_{j}\right)}-CCI_{i}{}^{\left(S_{j}\right)}\right|+TEMP_{i-1}{}^{\left(S_{j}\right)}$101: $Norm\_TEMP_{i}{}^{\left(S_{j}\right)}\leftarrow$
$TEMP_{i}{}^{\left(S_{j}\right)}/100$11: **End for**12: $AMoF_{i}{}^{\left(S_{j}\right)}$← $Norm\_TEMP_{i}{}^{\left(S_{j}\right)}/n+\;AMoF_{i}{}^{\left(S_{j}\right)}$13: **End for**14: Receive $AMoF^{\left(NUT\right)}{}_{1}$,..,$AMoF^{\left(NUT\right)}{}_{N-1}$ S.T $AMoF^{\left(NUT\right)}$ is  l×(N−1)15: **For**
*k* = 1 to N−1
**do**16: **For**
*j* = 1 to *l*
**do**17: **If**
*k* ≥ 2 **then**18: Find $\beta_{k}$ by solving (1)19: Find $C_{k}$ by solving (2)20: Find time varying threshold $\delta_{k}$ = $\left(\frac{max\left(C_{k}\right)-min\left(C_{k}\right)}{2}\right)+min\left(C_{k}\right)$21: @k=322: $\delta \leftarrow \delta_{k}$23: **If**
$\delta_{k}>\delta$24: Node is normal25: **Else**26: Node is malicious27: **End for**28: **End for**

## 4. Blackhole and DDoS Attack

In this section two types of malicious activities deployed in the experiments are described; blackhole attack and DDoS (flooding).

(a) Blackhole attack

The blackhole attack adopted in this work is based on [29], where a malicious node forges a fake route reply (RREP) that contains misleading information about its sequence number, the smaller the sequence number the fresher is the path, promoting him as the node having the shortest path to the destination node.

(b) DDoS (flooding)

It is a denial of service (DoS) based malicious activity which causes a disruption during the functioning of the network, by flooding the network with redundant data. In this paper, the route request (RREQ) flooding attack is used to simulate flooding activity [30]. DDoS is achieved by sending a large volume of traffic through the network which might lead to exhausting the network resources, overall bandwidth, and individual node resources.

## 5. Experimental Setup

Two extreme scenarios were tested based on node velocity and power level. Those scenarios are abbreviated as: NS1P3 and NS15P7 which refers to node velocity 1 m/s with power level of 3 dBm, and node velocity 15 m/s with power level of 7 dBm, respectively. The reason was to test the performance of the IDS under extreme connectivity levels. The connectivity is the lowest at NS1P3 and highest at NS15P7 [31]. 

**Figure 2:**
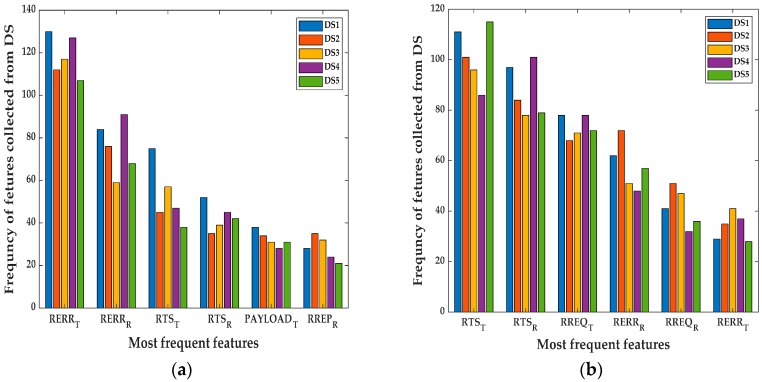
The most frequent features counted over all reporting times for the blackhole and flooding for both NS15P7 and NS1P3 scenarios: (**a**) Most frequent features in the blackhole case; (**b**) most frequent features in the flooding case.

The initial set of features used in the experiment are shown in Table 1. Those 12 features are collected from both MAC and network layer. A correlation-based attribute evaluator [32], is used to pick the most significant features based on their weight. The highest six frequent features that appeared in both NS1P3 and NS15P7 scenarios collected over the *Tr*, are shown in Figure 2. It is important to mention that those features are not optimum for the detection process, it presents some degree of redundancy which acts as noise. This is meant to test the IDS under suboptimal situations.

The data sets were generated by simulating a network with 30 nodes over an area of 1000 m^2^ over 2000 s period. The network profile when no malicious activity is generated over 20 different seeds. The same procedure is applied when generating the malicious activity for blackhole attack and the flooding attack with designating three malicious nodes in each case. The flooding attack is based on RREQ. Two different mobility models are adopted in this paper, the RWP which is the benchmark for all mobility models and the GM which offers temporal correlation for the node’s velocity. A memory value (α) is chosen equal to 0.5. It is a midpoint between a memoryless state where node’s velocity at each time slot has no correlation (such as the RWP), and strong memory case where node’s velocity at time slot is exactly as the pervious velocity [33].

The basic set of features used in the detection process are shown in Table 1 which will be reduced as mentioned before to six features for each type of attack. For the blackhole attack, the most frequent features obtained using the correlation-based attribute evaluator are: Route error transmitted (RERR_T_), route error received (RERR_R_), request-to-send transmitted (RTS_T_), request-to-send received (RTS_R_), PAYLOAD_T_, RREP_R_ as shown in Figure 2a. The most frequent features in the case of flooding attack are: RTS_T_, RTS_R_, RREQ_T_, RERR_R_, RREQ_R_, RERR_T_ as shown in Figure 2b.

The power levels, the node’s mobility, and other simulation parameters are listed in Table 2. Notice that the total reporting points in the experiment are: Simulation time/*Tr* = 2000/25 s = 80.

## 6. Results and Discussion

In this section, the results are presented for extreme node velocities 1 and 15 m/s, and for the extreme power level 3 and 7 dBm. This represents in abbreviated form NS1P3 and NS15P7. Both scenarios are tested under blackhole (BH) and flooding (FL) attacks with both mobility models RWP, GM. Reporting time (*Tr*) is 25 s and sampling time (*Ts*) is 5 s. The detection parameters are true positive rate (TPR) also called recall, true negative rate (TNR), false positive rate (FPR), and false negative rate (FNR), precision, and F_1_ score are shown in Equations (3)–(8). A detailed explanation for the detection performance using these equations and Algorithm 1, will be presented in the discussion section.

(3) $$TPR=\frac{TP}{TP+FN}$$

(4) $$TNR=\frac{TN}{TN+FP}$$

(5) $$FPR=\frac{FP}{FP+TN}$$

(6) $$FNR=\frac{FN}{FN+TP}\;$$

(7) $$Precision=\frac{TP}{TP+FP}\;$$

(8) $$F_{1}=2*\frac{Precision.Recall}{Precision+Recall}\;$$

Based on [34], the TP, FN, FP, and TN are defined as:

True positive (TP): Represents the number of malicious nodes that have been correctly classified as malicious.

False negative (FN): Represents the number of malicious nodes that have been misclassified as benign nodes.

False positive (FP): Represents the number of benign nodes that have been misclassified as malicious.

True negative (TN): Represents the number of benign nodes that have been correctly classified as benign.

An example showing how the results were obtained based on Equations (3)–(8) and Algorithm 1 is shown below. Every fitted slope point has a lower bound (LB) and upper bound (UB), malicious nodes reside in the region below the threshold whereas the benign nodes reside in the region above the threshold. The errors arise from the fact that malicious nodes UBs pass the threshold towards the benign nodes region, and the benign nodes LBs pass the threshold towards the malicious nodes region.

(1) TP = sum (UB (19) < threshold) + sum (LB (21) < threshold);

It counts the points related to the malicious nodes (19 and 21) which their upper bound points are less than the threshold. Since the malicious nodes have smaller slopes than the benign nodes.

(2) FP = sum (LB (13) < threshold) + sum (LB (23) < threshold);

It counts the points related to the benign nodes (13 and 23) which their lower bound points are less than the threshold.

(3) TN = sum (LB (13) > threshold) + sum (LB (23) > threshold);

It counts the points related to the benign nodes (13 and 23) which their lower bound points exceed the threshold.

(4) FN = sum (UB (19) > threshold) + sum (UB (21) > threshold);

It counts the points related to the malicious nodes (19 and 21) which their upper bound points exceed the threshold.

The performance of the IDS which is characterized by the: TPR, FPR, TNR, FNR, and the F_1_ score is presented in Table 3, Table 4, Table 5, Table 6, Table 7, Table 8, Table 9 and Table 10.

**Figure 3:**
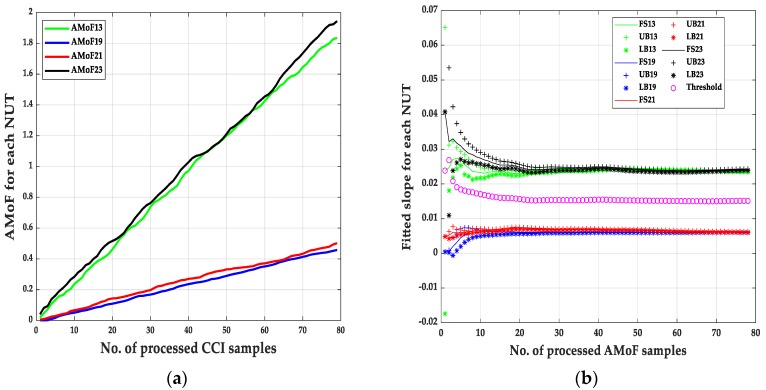
The AMoF and the fitted slope for different nodes for scenario NS15P7: (**a**) The AMoF for different NUT; (**b**) the fitted slope for NS15P7_FL_RWP 25/5.

**Figure 4:**
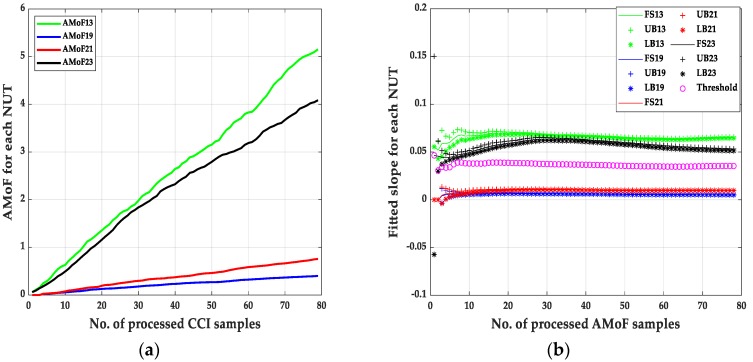
The AMoF and the fitted slope for different nodes for scenario NS15P7: (**a**) The AMoF for different NUT; (**b**) the fitted slope for NS15P7_BH_RWP 25/5.

**Figure 5:**
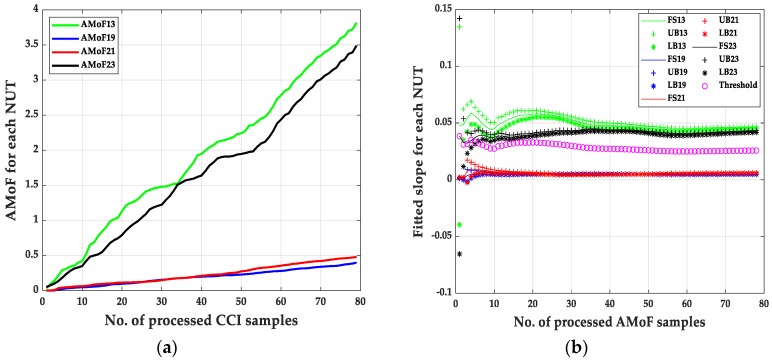
The AMoF and the fitted slope for different nodes for scenario NS15P7: (**a**) The AMoF for different NUT; (**b**) the fitted slope for NS15P7_FL_GM 25/5.

**Figure 6:**
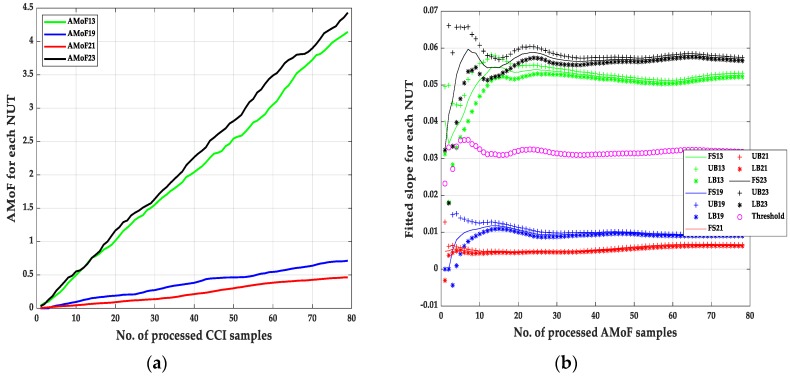
The AMoF and the fitted slope for different nodes for scenario NS15P7: (**a**) The AMoF for different NUT; (**b**) the fitted slope for NS15P7_BH_GM 25/5.

It is noticed that the IDS can identify the malicious nodes with a near perfect detection of different scenarios with TPR = 1 always, which show robustness in identifying malicious nodes with different deployment scenarios (power levels and node mobility). The main difference in the performance of the IDS showed up when identifying benign nodes as malicious nodes with FPR varies from 1.28% to 4.49% in the NS15P7 scenario, Figure 3, Figure 4, Figure 5 and Figure 6 and its corresponding tables, for RWP mobility and flooding attack in the first case and GM mobility and blackhole attack in the second case. This can be due to the nature of the blackhole attack, being more deceptive than the flooding attack since it does not just drop packets but tricks the designated traffic from source to destination to be forwarded through the malicious node. Moreover, the connectivity with RWP is better than GM which helps the data acquisition process that is needed to build the models for detection.

**Figure 7:**
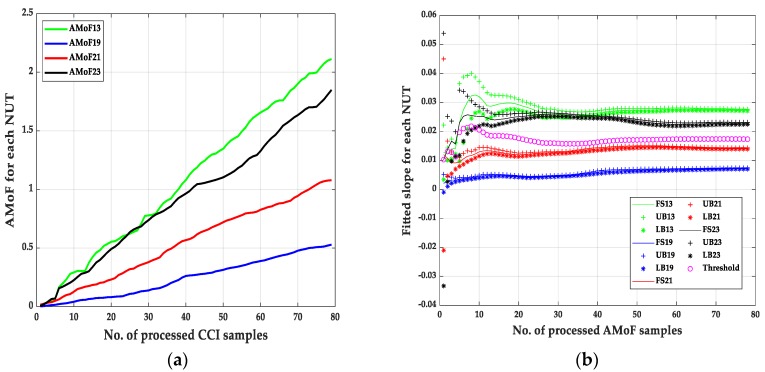
The AMoF and the fitted slope for different nodes for scenario NS1P3: (**a**) The AMoF for different NUT; (**b**) the fitted slope for NS1P3_FL_RWP 25/5.

**Figure 8:**
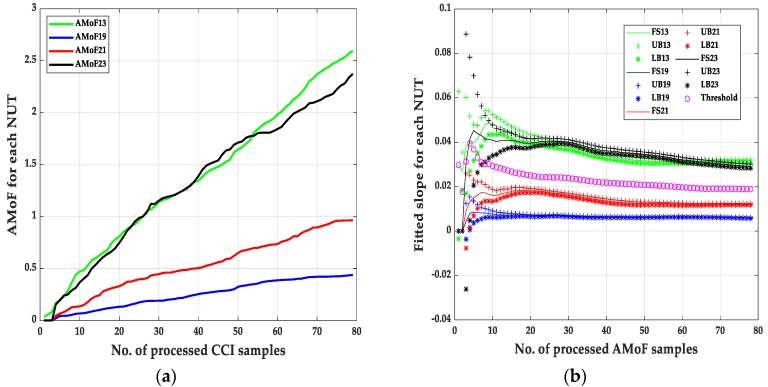
The AMoF and the fitted slope for different nodes for scenario NS1P3: (**a**) The AMoF for different NUT; (**b**) the fitted slope for NS1P3_BH_RWP 25/5.

**Figure 9:**
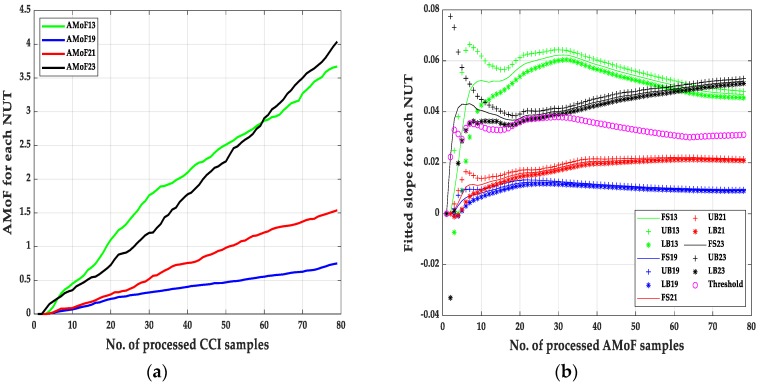
The AMoF and the fitted slope for different nodes for scenario NS1P3: (**a**) The AMoF for different NUT; (**b**) the fitted slope for NS1P3_FL_GM 25/5.

**Figure 10:**
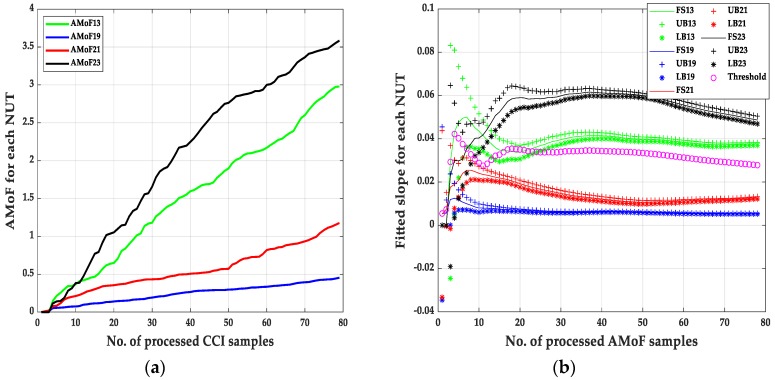
The AMoF and the fitted slope for different nodes for scenario NS1P3: (**a**) The AMoF for different NUT; (**b**) the fitted slope for NS1P3_BH_GM 25/5.

The second set of results are shown in Figure 7, Figure 8, Figure 9 and Figure 10 and its corresponding tables. These results are related to the NS1P3 scenario. A deterioration in the FNRs is noticed, nearly three times as it results in NS15P7. FNR varies between 10–12%, it is related directly to the IDS capability of collecting enough packets at such low connectivity, leading to larger errors when compared to the NS15P7 scenario.

The F_1_ score obtained for all the tested scenarios is above 90%. A highest F_1_ score of 99.36% is obtained for the NS15P7 scenario under the DDoS attack with the RWP mobility. The lowest F_1_ score of 93.94% is obtained for the NS1P3 scenario under the blackhole attack with GM mobility.

It is important to mention that choosing the location of the threshold, which is at the third iteration, has a significant effect on the results especially when dealing with the NS1P3 scenario. Notice the fluctuating nature of the fitted slopes figures, which makes choosing a proper location for the detection threshold, a hard task compared to the NS15P7 scenario.

## 7. Conclusions

An extended study based on previous work for a multistage cross layer-based IDS is presented. A robust IDS is presented and tested under extreme deployment scenarios (power levels and node’s velocity). Detection rates (TPR) were near perfect in most of the scenarios presented. F_1_ score varied between 93% and 99.36%. However, the limitation to this IDS is the false positive (FPR), which varied between 1.3% and 12% across various scenarios. The detection process is affected at the early stages of the fitted slope calculation. This is mainly due to the lack of packet counts that is related to the features used in the detection process. This problem mostly appears in the lower connectivity scenario, the NS1P3. A possible solution can be based on filtering these early stages of the fitted AMoF points.

Using a more complicated technique, based on the adaptive feature selection process at each reporting time, is another way to improve the performance and provide better differentiation between benign and malicious nodes during the early stages of the fitted slope process.

## Figures and Tables

**Table 1:** Cross layer features.

**Mac layer**	Tx/Rx	Tx/Rx	Tx/Rx
	RTS	CTS	ACK
**Network layer**	Tx/Rx	Tx/Rx	Tx/Rx
	RREQ	RREP	RERR

**Table 2:** Simulation parameters.

**No. of Nodes**	30
**Field area**	1000 × 1000 m
**Node speed**	1 and 15 m/s
**Simulation time**	2000 s
**Power levels**	3 and 7 dBm
**Routing protocol**	AODV
**Mobility model**	RWP, GM
**Reporting time (*Tr*)**	25 s
**Sampling time (*Ts*)**	5 s

**Table 3:** Performance characterization for NS15P7_FL_RWP 25/5.

TPR	FPR	TNR	FNR	F_1_
1	0.0128	0.9872	0	0.9936

**Table 4:** Performance characterization for NS15P7_BH_RWP 25/5.

TPR	FPR	TNR	FNR	F_1_
1	0.0192	0.9808	0	0.9905

**Table 5:** Performance characterization for NS15P7_FL_GM 25/5.

TPR	FPR	TNR	FNR	F_1_
1	0.0321	0.9679	0	0.9842

**Table 6:** Performance characterization for NS15P7_BH_GM 25/5.

TPR	FPR	TNR	FNR	F_1_
1	0.0449	0.9551	0	0.9781

**Table 7:** Performance characterization for NS1P3_FL_RWP 25/5.

TPR	FPR	TNR	FNR	F_1_
0.9936	0.1026	0.8974	0.0064	0.9483

**Table 8:** Performance characterization for NS1P3_BH_RWP 25/5.

TPR	FPR	TNR	FNR	F_1_
1	0.1218	0.8782	0	0.9426

**Table 9:** Performance characterization for NS1P3_FL_GM 25/5.

TPR	FPR	TNR	FNR	F_1_
1	0.1090	0.8910	0	0.9483

**Table 10:** Performance characterization for NS1P3_BH_GM 25/5.

TPR	FPR	TNR	FNR	F_1_
0.9568	0.0833	0.9167	0.0432	0.9394

## Appendix A

In this appendix, a list of terms mentioned in this work are listed in Table A1.

**Table A1:** List of abbreviations mentioned in this paper.

Term	Meaning
NS1P3	Node velocity 1 m/s, power level 3 dBm
NS15P7	Node velocity 15 m/s, power level 7 dBm
GM	Gauss Markov mobility model
SN	Super node
DS	Dedicated sniffer
RWP	Random way point mobility model
BH	Blackhole attack
FL	Flooding attack
*Tr*	Reporting time
*Ts*	Sampling time
FS	Fitted slope
UB	Upper bound
LB	Lower bound
TPR	True positive rate
FPR	False positive rate
TNR	True negative rate
FNR	False negative rate
RTS	Request-to-send
CTS	Clear-to-send
ACK	Acknowledgement
RREQ	Route request
RREP	Route reply
RERR	Route error
NS15P7_FL_RWP 25/5	Scenario with corresponding node velocity of 15 m/s, power level of 7 dBm, attack type flooding, mobility model RWP, and reporting/sampling time of 25/5 s.
NS15P7_BH_RWP 25/5	Scenario with corresponding node velocity of 15 m/s, power level of 7 dBm, attack type blackhole, mobility model RWP, and reporting/sampling time of 25/5 s.
NS15P7_FL_GM 25/5	Scenario with corresponding node velocity of 15 m/s, power level of 7 dBm, attack type flooding, mobility model GM, and reporting/sampling time of 25/5 s.
NS15P7_BH_GM 25/5	Scenario with corresponding node velocity of 15 m/s, power level of 7 dBm, attack type blackhole, mobility model GM, and reporting/sampling time of 25/5 s.
NS1P3_FL_RWP 25/5	Scenario with corresponding node velocity of 1 m/s, power level of 3 dBm, attack type flooding, mobility model RWP, and reporting/sampling time of 25/5 s.
NS1P3_BH_RWP 25/5	Scenario with corresponding node velocity of 15 m/s, power level of 7 dBm, attack type blackhole, mobility model RWP, and reporting/sampling time of 25/5 s.
NS1P3_FL_GM 25/5	Scenario with corresponding node velocity of 15 m/s, power level of 7 dBm, attack type flooding, mobility model GM, and reporting/sampling time of 25/5 s.
NS1P3_BH_GM 25/5	Scenario with corresponding node velocity of 15 m/s, power level of 7 dBm, attack type blackhole, mobility model RWP, and reporting/sampling time of 25/5 s.

## References

[1] Mishra A., Sudan K., Soliman H. Detecting Border Intrusion Using Wireless Sensor Network and Artificial Neural Network. Proceedings of the 6th IEEE international conference on distributed computing in sensor systems workshops (DCOSSW).

[2] Diro A.A., Chilamkurti N. (2018). Distributed Attack Detection Scheme Using Deep Learning Approach for Internet of Things. Future Gener. Comput. Syst..

[3] Kaplantzis S., Shilton A., Nallasamy M., Sekercioglu Y. Detecting Selective Forwarding Attacks in Wireless Sensor Networks Using Support Vector Machines. Proceedings of the 3rd IEEE International Conference on Intelligent Sensors, Sensor Networks and Information.

[4] Amouri A., Jaimes L.G., Manthena R., Morgera S.D., Vergara-Laurens I.J. A simple scheme for pseudo clustering algorithm for cross layer intrusion detection in MANET. Proceedings of the 7th IEEE Latin-American Conference on Communications (LATINCOM).

[5] Sutharshan R., Leckie C., Palaniswami M., Bezdek J.C. (2008). Anomaly Detection in Wireless Sensor Networks. IEEE Wirel. Commun..

[6] Amor N., Benferhat S., Elouedi Z. Naive Bayes vs Decision Trees in Intrusion Detection Systems. Proceedings of the 2004 ACM symposium on Applied computing.

[7] Lim T.-S., Loh W.-Y., Shih Y.-S. (2000). A comparison of prediction accuracy, complexity, and training time of thirty-three old and new classification algorithms. Mach. Learn..

[8] Amouri A., Morgera S., Bencherif M., Manthena R. (2018). A Cross-Layer, Anomaly-Based IDS for WSN and MANET. Sensors.

[9] Panhong W., Shi L., Wang B., Wu Y., Liu Y. Survey on Hmm Based Anomaly Intrusion Detection Using System Calls. Proceedings of the IEEE 5th International Conference on Computer Science & Education.

[10] Constantinos K., Kambourakis G., Maragoudakis M. (2011). Swarm Intelligence in Intrusion Detection: A Survey. Comput. Secur..

[11] Shahid R., Wallgren L., Voigt T. (2013). Svelte: Real-Time Intrusion Detection in the Internet of Things. Ad Hoc Netw..

[12] Alaparthy V.T., Amouri A., Morgera S.D. (2018). A Study on the Adaptability of Immune Models for Wireless Sensor Network Security. Procedia Comput. Sci..

[13] Alaparthy V.T., Morgera S.D. (2018). A Multi-Level Intrusion Detection System for Wireless Sensor Networks Based on Immune Theory. IEEE Access.

[14] Alaparthy V., Morgera S.D. (2019). Modeling an Intrusion Detection System Based on Adaptive Immunology. Int. J. Interdiscip. Telecommun. Netw..

[15] Amouri A., Alaparthy V.T., Morgera S.D. Cross Layer-Based Intrusion Detection Based on Network Behavior for IoT. Proceedings of the 19th IEEE Wireless and Microwave Technology Conference (WAMICON).

[16] Amouri A. (2019). Cross Layer-Based Intrusion Detection System Using Machine Learning for MANETs.

[17] Hongmei D., Zeng Q.A., Agrawal D. SVM-Based Intrusion Detection System for Wireless Ad Hoc Networks. Proceedings of the IEEE 58th Vehicular Technology Conference.

[18] Cabrera J., Gutiérrez C., Mehra R. Infrastructures and Algorithms for Distributed Anomaly-Based Intrusion Detection in Mobile Ad-Hoc Networks. Proceedings of the IEEE Military Communications Conference.

[19] Cabrera J., Gutiérrez C., Mehra R. (2008). Ensemble Methods for Anomaly Detection and Distributed Intrusion Detection in Mobile Ad-Hoc Networks. Inf. Fusion.

[20] Kurosawa S., Nakayama H., Kato N., Jamalipour A., Yoshiaki N. (2007). Detecting Blackhole Attack on Aodv-Based Mobile Ad Hoc Networks by Dynamic Learning Method. Int. J. Netw. Secur..

[21] Bose S., Bharathimurugan S., Kannan A. Multi-Layer Integrated Anomaly Intrusion Detection System for Mobile Adhoc Networks. Proceedings of the IEEE International Conference on Signal Processing, Communications and Networking.

[22] Mitrokotsa A., Komninos N., Douligeris C. Intrusion Detection with Neural Networks and Watermarking Techniques for Manet. Proceedings of the IEEE International Conference on Pervasive Services.

[23] Mitrokotsa A., Dimitrakakis C. (2013). Intrusion Detection in Manet Using Classification Algorithms: The Effects of Cost and Model Selection. Ad Hoc Netw..

[24] Azmoodeh A., Dehghantanha A., Choo K.K.R. (2018). Robust Malware Detection for Internet of (Battlefield) Things Devices Using Deep Eigenspace Learning. IEEE Trans. Sustain. Comput..

[25] Doshi R., Apthorpe N., Feamster N. Machine Learning DDoS Detection for Consumer Internet of Things Devices. Proceedings of the IEEE Security and Privacy Workshops (SPW).

[26] Thamilarasu G., Chawla S. (2019). Towards Deep-Learning-Driven Intrusion Detection for the Internet of Things. Sensors.

[27] Sterne D., Balasubramanyam P., Carman D., Wilson B., Talpade R., Ko C., Balupari R., Tseng C.-Y., Bowen T. A general cooperative intrusion detection architecture for MANETs. Proceedings of the Third IEEE International Workshop on Information Assurance.

[28] Draper N.R., Smith H. (1998). Fitting a straight line by least squares. Applied Regression Analysis.

[29] Ehsan H., Khan F.A. Malicious AODV: Implementation and Analysis of Routing Attacks in Manets. Proceedings of the IEEE 11th International Conference on Trust, Security and Privacy in Computing and Communications.

[30] Alokparna B., Vuppala S., Choudhury P. A Simulation Analysis of Flooding Attack in Manet Using NS-3. Proceedings of the IEEE 2nd International Conference on Wireless Communication, Vehicular Technology, Information Theory and Aerospace & Electronic Systems Technology (Wireless VITAE).

[31] Chu T., Nikolaidis I. (2004). Node density and connectivity properties of the random waypoint model. Comput. Commun..

[32] Hall M.A. (1999). Correlation-Based Feature Selection for Machine Learning.

[33] Bai F., Helmy A. (2006). A Survey of Mobility Models in Wireless Ad-Hoc Networks. Wirel. Ad Hoc Sens. Netw..

[34] Detection Accuracy. https://www.sciencedirect.com/topics/computer-science/detection-accuracy.

